# The Efficacy and Safety of Rituximab in a Patient with Rheumatoid Spondylitis

**DOI:** 10.1155/2013/792526

**Published:** 2013-11-12

**Authors:** Şenol Kobak, Ahmet Karaarslan, Fahrettin Oksel

**Affiliations:** ^1^Department of Rheumatology, Faculty of Medicine, Şifa University, Bornova, 35100 Izmir, Turkey; ^2^Department of Orthopaedic Surgery, Faculty of Medicine, Şifa University, Bornova, 35100 Izmir, Turkey; ^3^Department of Rheumatology, Faculty of Medicine, Ege University, 35100 Izmir, Turkey

## Abstract

Rheumatoid arthritis (RA) is considered as a connective tissue disease while ankylosing spondylitis (AS) is a prototype of spondyloarthritis. These diseases are seen concomitantly only very rarely. Also, rituximab has proven efficacy in the treatment of RA while its role in the treatment of AS is unclear. In this presentation, the concomitant presence of RA and AS in a 43-year-old male patient as well as the efficacy and safety of rituximab is discussed. Rituximab was given due to lack of response to treatment with anti-TNF-alpha. Evaluations made at the 6th and 12th months of treatment showed complete response for RA and partial response for AS.

## 1. Introduction

Ankylosing spondylitis (AS) is a chronic inflammatory disease which primarily affects the sacroiliac joint and axial and peripheral joints [1]. It should typically be considered in young male patients with complaints of inflammatory lower back pain. Rheumatoid arthritis (RA), however, is a connective tissue disease which can lead to erosive arthritis and results in significant morbidity and mortality [2]. These diseases have different mechanisms of etiopathogenesis and they are rarely seen in the same person. Although the term “rheumatoid spondylitis” is used to describe the cervical involvement of RA, it can also be used for people who have both diseases concomitantly. Different disease-modifying antirheumatic drugs (DMARDs) are used for the treatment of these diseases [3]. The efficacy and safety of rituximab (RTX), in addition to DMARDs and anti-TNF-alpha drugs, have been proved in the treatment of RA. RTX is a chimeric monoclonal antibody targeted against CD20 which is used in RA patients who do not respond to treatment with one or more anti-TNF-alpha drugs [4]. Various studies have shown that it can be effective in other autoimmune diseases too [5, 6]. Anti-TNF-alpha drugs have revolutionized the treatment of AS by shaping the course and prognosis of the disease in the axial and peripheral involvement of AS [7]. However, disease activity canot be controlled with TNF-blockers in 50% of AS patients. Therefore, other treatment options are considered urgently in cases where anti-TNF-alpha drugs do not lead to a response and/or are contraindicated. Histological and MRI studies showed that the primary area of inflammation in AS is the cartilage and bone surface [8]. Mononuclear cell infiltration was detected in the cartilage and subchondral bone. Infiltrations, primarily of macrophages and T-cells, with specific immune response were identified in early and active sacroiliitis [9]. Surprisingly, B-cell infiltrations were also found in the subchondral bone marrow of these patients. There was a significant increase of CD20+ B-cells in the biopsy material collected from AS patients during spinal surgery and these findings were associated with MRI images [10]. In addition to such immunohistological data, its proven efficacy in RA patients suggests that RTX can also be an effective treatment option in AS patients. There is limited data in the literature on the use of RTX in AS patients. The safety and efficacy of RTX in a 43-year-old male patient diagnosed with rheumatoid spondylitis are reported in this presentation.

## 2. Case Report

The 43-year-old male patient started to have complaints of joint pain and restricted mobility in the right hip, inflammatory lower back pain, and morning stiffness in 1987. The patient visited several doctors and was given anti-inflammatory therapy only. Two years after the onset of complaints, he was diagnosed with ankylosing spondylitis as a result of the tests made by his rheumatologist. The patient was started on corticosteroid (CS) 4 mg/day, methotrexate (MTX) 15 mg/day, sulfasalazine (SSZ) 2 g/day, and NSAID. When this treatment provided remission of his complaints, he discontinued his drugs and failed to return for control visits from time to time. In 1997 he underwent total hip replacement and infliximab was first started in 2004 due to active disease. The patient remained on this treatment until 2006, when his complaints worsened and etanercept was started after disease activation was identified in the tests. Having used etanercept for the last 5 years, the patient presented to our polyclinic in January 2011. He had inflammatory lower back pain, morning stiffness lasting until noon, pain and restricted mobility in both wrists and ankles, and arthritis in the right knee joint despite receiving therapy. First evaluation revealed findings of AS posture, pain and tenderness in both wrists and ankles, ulnar deviation in the MCF joints of hands, tenderness and deformities in ankles, and synovitis in the right knee joint. Systemic examination was normal. Measurements made for AS gave the following results: finger-to-floor distance: 8 cm, occiput-to-wall distance: 3 cm, Schober test: 2 cm, chest expansion: 3 cm, chin-to-sternum distance: 2 cm. With regard to disease activation, BASFI and BASDAI tests gave a score of 7 cm and 7.8 cm, respectively.

The laboratory tests gave the following results: erythrocyte sedimentation rate (ESR): 98 mm/h, C-reactive protein (CRP): 14.3 mg/dL (normal: 0–0.8), rheumatoid factor (RF): negative, anti-CCP: 41 IU/mL (normal <5), ANA, anti-Ro, and anti-La antibodies: negative, serum amyloid A protein: 1260 mg/L (normal <7 mg/L). Polyclonal gammopathy was detected by protein electrophoresis. HLA-B27 was positive. Liver and renal function tests and urinalysis were normal. An abdominal ultrasonography and chest X-ray were performed and found normal. X-ray of the sacroiliac joint (SIJ) showed that the SIJ was bilaterally closed and ankylosed. Cervical and thoracolumbar radiography revealed a “bamboo spine.” Periarticular osteoporosis, subchondral sclerosis, and sporadic erosions were detected in wrist and ankle X-rays. Ankle MR images showed deformed bones, narrowed joint space in tibiotalar, talocrural, subtalar, calcaneocuboid, and cuneonavicular joints, and irregularities, subchondral sclerosis, resorption cysts, and erosion on joint surfaces. These findings were consistent with RA. Thoracic CT was normal except for a reactive LAP of mm in the mediastinal and axillary areas. Abdominal CT showed suspicious wall thickening in the lateral gastric antrum and a LAP of 2 × 1.5 cm in the bilateral inguinal region. Having received anti-TNF-alpha treatment for a long time, the patient was evaluated for possible malignancy and/or specific infection and the anti-TNF-alpha drug was discontinued. An upper GI endoscopy was performed and erythematous antral gastritis was identified. The biopsies collected from here were benign. An evaluation was also made for amyloidosis; Congo red staining was negative. The patient was evaluated for a possible lymphoma; there were no B symptoms, and no progression was seen compared to the abdominal CT performed 6 months earlier and LAPs were reactive. So biopsy was not considered and followup was recommended. Evaluating the clinical and laboratory findings, the case was considered to be RA concomitant with AS. The patient's complaints improved following the arthrocentesis and local steroid administration in the left knee joint for synovitis. The joint puncture fluid showed inflammation; there was no bacteria growth including ARB. The patient had active disease despite taking traditional DMARDs and anti-TNF-alpha and it was planned to give him RTX in addition to MTX. After obtaining a consent form from the patient, he was given RTX 1000 mg (2nd dose after 15 days) as i.v. infusion. The second RTX infusion was administered 6 months later. The evaluation made at the 6th month of therapy showed remission of his complaints. Laboratory tests showed no pathological findings other than the following: ESR: 45 mm/h and CRP: 3.5 mg/dL. In the evaluation made at the 1st year of therapy, it was seen that there was a reduction in acute phase response (ESR, CRP) reactants, but the values were not completely normal (Table 1). When pre- and posttreatment SIJ MRI findings were compared, it was seen that the bone edema had resolved, but there was no change in chronic findings. No change was observed in AS-related measurement parameters (Schober, chest expansion, etc.). BASDAI and BASFI scores had declined, but there was no complete response. When inquired, the patient stated that his inflammatory lower back pain had improved and morning stiffness lasted shorter than one hour. In light of such data, it was considered that rituximab resulted in a complete response for RA and a partial response for AS. RTX therapy was tolerated well with no major and/or minor side effects.

## 3. Discussion

The safety and efficacy of RTX in a rheumatoid spondylitis case with resistance to anti-TNF-alpha treatment are discussed in this presentation. Response was achieved in all parameters evaluated for RA while there was a partial response for AS activity indices. There was improvement in active lesions in the SIJ MR findings, but chronic changes persisted. There was no regression in any of the measurements (Schober, etc.). The efficacy of RTX in RA has been shown, but the data on AS patients are limited and inconsistent. Song et al. investigated the efficacy of RTX in 20 AS patients who did not respond to treatment with TNF-alpha or were naïve to TNF-alpha [11]. The evaluation made at the end of 24 weeks showed that RTX was not effective in patients who did not respond to TNF-alpha before but was an effective treatment option for patients who were naïve to TNF-alpha. Several case reports in the literature on the efficacy of RTX in AS also have inconsistent results. Rodríquez-Escalera et al. gave RTX therapy to anti-TNF-alpha-naïve AS and chronic hepatitis B patients [12]. There was a marked response in the disease activation parameters followed and no HBV reactivation. According to the data of the French Rheumatology Society, the efficacy of RTX was evaluated retrospectively in 26 SpA patients [13]. Overall, there was response in 11 patients; anti-TNF-alpha-naïve patients [3] responded well to therapy while 8 patients with anti-TNF-alpha resistance gave partial response. Generally and interestingly, AS patients who did not respond to TNF-alpha-blockers did not respond to RTX therapy either in other studies. Such cases include a female patient who had active disease despite using 3 TNF-blockers, a male patient who did not respond to etanercept therapy, and another female patient with partial response to the same [14, 15]. On the other hand, according to another study RTX resulted in very good response in 3 AS patients who could not use TNF-alpha-blockers due to contraindications [16]. According to the literature and similar to our case, while AS patients with no response to previous therapy with TNF-blockers did not respond to RTX therapy either, very good outcomes were achieved in TNF-alpha-naïve patients. These results have resulted in a questioning of possible mechanisms regarding B-cell-targeting treatments in AS. The efficacy of RTX can be explained with reduced synthesis of certain proinflammatory cytokines (TNF-alpha, IL-1, and IL-6) due to depletion of CD20 and/or interaction with other inflammatory pathways. As known, there is yet no evidence for the presence of any autoantibody in the pathogenesis of AS. B-cells are also potent antigen-presenting cells and inhibition of this function can be an alternative explanation. Another view claims that both TNF-alpha-blockers and anti-B-cell therapies inhibit similar pathways. Response to therapy can be associated not only with the mechanisms of action of the drugs used but also with the stage and duration of disease, use of other drugs, and/or genetic factors. The role of B-cells in the immune response given in AS and the results of immunohistological studies strongly suggest that RTX can be an effective therapy option [17]. In light of this information, RTX can become an alternative treatment option in the near future for AS patients for whom TNF-blockers are contraindicated, for example, because of TBC, lymphoma, or demyelinating disease. The positive results obtained in anti-TNF-alpha-naïve patients should be supported with placebo controlled studies and/or studies that compare RTX and TNF-blocker drugs. In conclusion, RTX is one of the important and effective therapy options in RA patients. In AS cases, on the other hand, it is thought that it can be an effective treatment modality in anti-TNF-alpha-naïve patients. New studies are warranted on this subject to support the limited data in the literature.

## Figures and Tables

**Table 1:** Clinical and laboratory findings in rheumatoid spondylitis patient before and after rituximab therapy.

	Baseline	6 months	12 months
Tender joint count	15	6	2
Swollen joint count	8	1	0
BASDAI, 0–10 cm	7.8	5.6	4.2
BASFI, 0–10 cm	7.0	6.4	4.7
DAS28	5.4	3.5	2.02
Schober test (cm)	2	2	2
Occiput-wall distance (cm)	3	3	3
Chest expansion (cm)	3	3	3
Finger-floor distance (10 cm)	8	9	12
VAS patient global health, 0–100 mm	95	54	33
VAS physician's global assessment	90	45	40
ESR, mm/h	98	45	38
CRP, mg/dL (normal value 0–0.5 mg/dL)	14.3	3.5	0.1

BASDAI: Bath Ankylosing Spondylitis Disease Activity Index.

BASFI: Bath Ankylosing Spondylitis Functional Index.
